# Cholera outbreak in Funpur, Winland

**DOI:** 10.11604/pamj.supp.2021.40.2.30989

**Published:** 2021-12-16

**Authors:** Muhammad Nadeem Shah, Esra Saleh, Eva Mertens, Hedia Bellali, Elizeus Rutebemberwa

**Affiliations:** 1 National Emergency Operation Center, Pakistan,; 2 Sudanese American Medical Association, Sudan,; 3 Bernhard Nocht Institute for Tropical Medicine, Department of Infectious Disease Epidemiology, Bernhard-Nocht-Strasse 74, 20359 Hamburg, Germany,; 4 Global Partnership Initiated Biosecurity Academia for Controlling Health Threats (GIBACHT), Hamburg, Germany,; 5 Habib Thameur Hospital, Department of Clinical Epidemiology, Tunis, Tunisia,; 6 Medical Faculty of Tunis, Tunis El Manar University, Tunis, Tunisia,; 7 Programs, African Field Epidemiology Training Network, Kampala, Uganda; 8 Makerere University College of Health Sciences, Kampala, Uganda,; 9 Global Partnership Initiated Biosecurity Academia for Controlling Health Threats (GIBACHT), Kampala, Uganda

**Keywords:** Man-made outbreak, biosafety, biosecurity, International Health Regulation

## Abstract

Vibrio Cholerae is a category B agent which has moderate to high potential to be used in bioterrorist events. This fictitious case study is based on man-made outbreak investigation and response carried out by disease surveillance and response unit of country Winland. The numbers of acute watery diarrhoea cases (AWDs) were concentrated in city Funpur of country Winland which share international border with Robiland, another country with poor health infrastructure. Regular movement of nomadic population between two countries has additional risk of international spread. This case study is designed for the training of public health students and workers on steps of outbreak investigation, packaging of biological samples, understanding IHR reporting algorithm, understanding difference between biosafety and biosecurity, different categories of bioterrorism organisms and PPE & its zones. This case study can be used as supporting training tool for application of learned concepts to a real situation and can be carried out in 2-3 hours.

## How to use this case study

### General instructions

This case study is a supporting teaching training tool designed for application of learned concepts to a real situation. This Case study is to be used for beginner and intermediate level trainees. During teaching sessions, the trainees can be divided into smaller groups of 5-8 persons, and each person can be asked by a facilitator to sub-facilitate a particular section. The facilitator needs to guide the overall discussion and bring discussion back on track, through questions, whenever the group loses the direction. He/She may also use flip charts to explain certain points.

### Target audience

The study aims to target all public health workers with beginner to intermediate level training. Field Epidemiologists, Environmental Health Officers, Laboratory Scientists, Health Educationists and Epidemiology students are key public health officials to be targeted.

### Pre-requisites

Participants need to be aware of disease surveillance and outbreak investigations concepts.

### Materials needed

Pen, papers, markers and Flip Papers/Charts

### Time required

2-3 hours

### Language

English

## Case study material

- Download the case study student guide (PDF - 876 KB)
- Request the case study facilitator guide: contact info@gibacht.org
